# Histomolekulare Klassifikation des Urothelkarzinoms der Harnblase: Vom histologischen Phänotyp zum Genotyp und zurück

**DOI:** 10.1007/s00292-024-01305-w

**Published:** 2024-01-29

**Authors:** Alexandra K. Stoll, Florestan J. Koll, Markus Eckstein, Henning Reis, Nadine Flinner, Peter J. Wild, Jochen Triesch

**Affiliations:** 1 Dr. Senckenbergisches Institut für Pathologie, Universitätsklinikum Frankfurt, Goethe-Universität Frankfurt, Theodor-Stern-Kai 7, 60590 Frankfurt am Main, Deutschland; 2 Frankfurt Institute for Advanced Studies (FIAS), Frankfurt am Main, Deutschland; 3 Klinik für Urologie, Universitätsklinikum Frankfurt, Frankfurt am Main, Deutschland; 4 Institut für Pathologie, Universitätsklinikum Erlangen, Friedrich-Alexander-Universität Erlangen-Nürnberg, Erlangen, Deutschland; 5 Frankfurt Cancer Institute (FCI), Frankfurt am Main, Deutschland; 6 University Cancer Center (UCT) Frankfurt-Marburg, Frankfurt am Main, Deutschland

**Keywords:** Urothelkarzinom, Künstliche Intelligenz, Molekulare Subtypen, Molekulare Heterogenität, The Cancer Genome Atlas, Urothelial carcinoma, Artificial intelligence, Molecular subtypes, Molecular heterogeneity, The Cancer Genome Atlas

## Abstract

**Hintergrund:**

Muskelinvasive Urothelkarzinome (MIUC) der Harnblase repräsentieren ca. 25 % aller Urothelkarzinome (UC) und weisen eine 5‑Jahres-Überlebensrate von ca. 50 % auf. Bisher haben Erkenntnisse aus der molekularen Klassifikation der MIUCs noch keinen Einfluss auf die klinische Praxis genommen.

**Ziel:**

Ziel der Arbeit ist die Vorhersage molekularer Konsensus-Subtypen in MIUCs mittels Künstlicher Intelligenz (KI) anhand histologischer Hämatoxylin-Eosin(HE)-Schnitte.

**Material und Methoden:**

Durchgeführt wurde ein pathologisches Review und die Annotation von Tumorarealen in der Bladder-Cancer(BLCA)-Kohorte (*N* = 412) des „The Cancer Genome Atlas“ (TCGA) und der BLCA-Kohorte (*N* = 181) des Dr. Senckenbergischen Instituts für Pathologie (SIP). Anhand der annotierten Histomorphologie zur Vorhersage molekularer Subtypen wurde ein KI-Modell trainiert.

**Ergebnisse:**

In einer 5fachen Kreuzvalidierung mit TCGA-Fällen (*N* = 274), internem TCGA-Testset (*N* = 18) und externem SIP-Testset (*N* = 27) erreichten wir durchschnittliche Werte der „area under the receiver operating characteristic curve“ (AUROC) von jeweils 0,73, 0,8 und 0,75 zur Klassifikation der verwendeten molekularen Subtypen *„luminal“, „basal/squamous“* und *„stroma-rich“*. Durch Training auf Korrelationen zu einzelnen molekularen Subtypen statt auf eine Subtypzuordnung pro Fall konnte die KI-Vorhersage von Subtypen signifikant verbessert werden.

**Diskussion:**

Nachfolgestudien mit RNA-Extraktion aus verschiedenen Bereichen KI-vorhergesagter molekularer Heterogenität könnten molekulare Klassifikationen und damit die darauf trainierten KI-Modelle verbessern.

**Zusatzmaterial online:**

Zusätzliche Informationen sind in der Online-Version dieses Artikels (10.1007/s00292-024-01305-w) enthalten.

## Abstract (en)

**Background:**

Of all urothelial carcinomas (UCs), 25% are muscle invasive and associated with a 5-year overall survival rate of 50%. Findings regarding the molecular classification of muscle-invasive urothelial carcinomas (MIUCs) have not yet found their way into clinical practice.

**Objectives:**

Prediction of molecular consensus subtypes in MIUCs with artificial intelligence (AI) based on histologic hematoxylin-eosin (HE) sections.

**Methods:**

Pathologic review and annotation of The Cancer Genome Atlas (TCGA) Bladder Cancer (BLCA) Cohort (*N* = 412) and the Dr. Senckenberg Institute of Pathology (SIP) BLCA Cohort (*N* = 181). An AI model for the prediction of molecular subtypes based on annotated histomorphology was trained.

**Results:**

For a five-fold cross-validation with TCGA cases (*N* = 274), an internal TCGA test set (*N* = 18) and an external SIP test set (*N* = 27), we reached mean area under the receiver operating characteristic curve (AUROC) scores of 0.73, 0.8 and 0.75 for the classification of the used molecular subtypes *“luminal”*, *“basal/squamous”* and *“stroma-rich”*. By training on correlations to individual molecular subtypes, rather than training on one subtype assignment per case, the AI prediction of subtypes could be significantly improved.

**Discussion:**

Follow-up studies with RNA extraction from various areas of AI-predicted molecular heterogeneity may improve molecular classifications and thereby AI algorithms trained on these classifications.

Ziel dieser Publikation ist es aufzuzeigen, wie eine Anwendung von KI-Algorithmen zur Vorhersage von molekularen Subtypen anhand von histologischen Hämatoxylin-Eosin(HE)-Schnitten des muskelinvasiven Urothelkarzinoms (MIUC) verbessert werden kann. Zudem werden die Hürden beleuchtet, die einer klinischen Anwendung der molekularen Subtypisierung – mit einem Fokus auf Datensätzen – noch im Wege stehen.

## Charakterisierung des Urothelkarzinoms

### Subtypen

Urothelkarzinome (UC) der Harnblase zeigen zusätzlich zur „typischen“ urothelialen Histologie („not otherwise specified“, NOS) eine histologische Vielfalt. UCs mit histologischen Subtypen identifizierte man in ca. 14 % aller PatientInnen mit invasivem UC, die sich einer radikalen Zystektomie (RZ) unterzogen hatten [4]. Invasive UCs mit squamöser Differenzierung sind einer der häufigsten histologischen Subtypen des UCs [22] und mit dem molekularen Subtyp „*basal/squamous“* („*Ba/Sq“*) assoziiert [8, 15], während z. B. der mikropapilläre Subtyp mit molekularen *„luminal“-Subtypen* assoziiert ist [6, 8]. In einer retrospektiven multizentrischen Kohortenstudie waren histologische Subtypen des UCs vermehrt mit fortgeschrittenem Tumorstadium, lymphovaskulärer Invasion und Lymphknotenmetastasen assoziiert [22]. Ein systematischer Review, der das Gesamtüberleben von PatientInnen nach radikaler Zystektomie untersuchte, zeigt, dass mikropapilläre und sarkomatoide Subtypen sowie reine neuroendokrine Tumoren der Harnblase mit einem geringeren Gesamtüberleben verbunden sind [14].

### Morphologie und Muskelinvasion

Morphologisch können UCs in papilläre, nichtpapilläre (solide) und gemischte Typen eingeteilt werden [7]. Der papilläre Typ ist am häufigsten und tritt überwiegend bei nichtinvasiven und in stromainvasiven UCs auf, die durch eine erhöhte Frequenz an *FGFR3*-Mutationen charakterisiert sind und sich selten zu muskelinvasiven UCs weiterentwickeln [3]. (Muskel-)invasive UCs, die überwiegend ein nichtpapilläres (solides) Wuchsmuster zeigen, sind häufig in den Genen *TP53* und *RB1* mutiert [21].

## Molekulare Subtypisierung des muskelinvasiven UC der Harnblase

### Molekulare Konsensus-Subtypen

**Figure 1:**
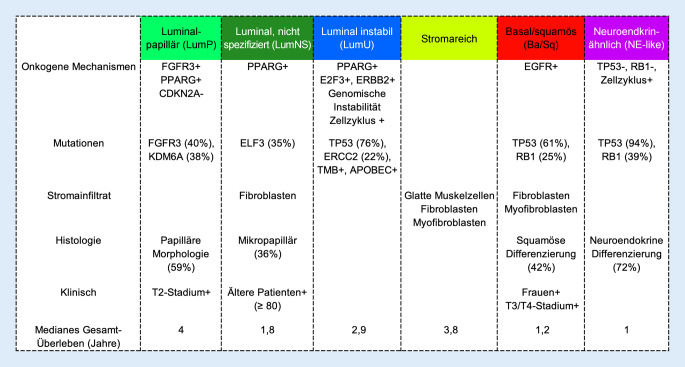

Verschiedene Forschergruppen – inklusive des „TCGA Cancer Research Network“ (TCGA, The Cancer Genome Atlas) – haben MIUCs anhand vergleichbarer molekularer Merkmale mithilfe von Bulk-RNA-Sequenzierung in Subtypen kategorisiert, die sich in Bezug auf Biologie, Prognose und Therapieansprechen unterscheiden [5]. Von diesen wurden 6 – einschließlich einer TCGA-Klassifikation [15] – anhand von 18 mRNA-Datensätzen durch Kamoun et al. in eine neue Konsensusklassifikation überführt [8]. Diese unterscheidet 6 molekulare Subtypen, die zum Teil bestimmte histologische Merkmale und Mutationen aufweisen und sich hinsichtlich Gesamtüberleben unterscheiden (Abb. 1).

### Molekulare Heterogenität kaum erfasst

In MIUCs können intratumoral verschiedene molekulare Subtypen gleichzeitig vorkommen, wobei Häufigkeit und prognostische Relevanz größtenteils unklar sind [19]. Da die Konsensusklassifikation und andere molekulare Klassifikationen meist auf RNA-Sequenzierung einer Tumorgewebeprobe basieren, kann so eine intratumorale molekulare Heterogenität nur unzureichend erfasst werden. Mittels multiregionaler Analyse mit immunhistochemischen Markern für molekulare Subtypen auf Grundlage von Sjödahl et al. [18] konnte gezeigt werden, dass in ca. einem von 4 MIUCs eine molekulare intratumorale Heterogenität vorliegt und dass diese häufiger in einem organbegrenztem (pT2) Stadium als in einem fortgeschrittenen Stadium (≥ pT3) vorkommt [16].

## Wissenschaftliche Fragestellung

Ziel war es, molekulare Konsensus-Subtypen von HE-gefärbten Schnitten basierend auf der Histologie von MIUCs mittels KI-Methoden vorherzusagen. Neben geringeren Kosten könnte ein solcher Ansatz – wenn er zuverlässig funktioniert – auch die intratumorale Heterogenität vorhersagen und sichtbar machen. Dies kann ebenfalls therapieentscheidend sein.

## Methodik

### Verwendete Datensätze

Für KI-Experimente wurde die TCGA-BLCA („bladder cancer“)-Kohorte verwendet [15]. Molekulare Konsensus-Subtypen übernahmen wir dafür von Kamoun et al. [8]. Zur Vereinfachung und Erhöhung der Fallzahlen pro Subtyp fassten wir Fälle mit *Luminal-non-specified‑, Luminal-papillary-* und *Luminal-unstable-Subtypen* zu einem gemeinsamen *Luminal-Subtyp* zusammen (Begründung für Zusammenfassung zu einem Subtyp siehe Onlinezusatzmaterial). Fälle mit einem *Neuroendocrine-like-Subtyp* wurden aus der Analyse ausgeschlossen. Als externes (ext.) Testset wurden MIUCs aus einer publizierten UC-Kohorte [11] des Dr. Senckenbergischen Instituts für Pathologie (SIP), Universitätsklinikum Frankfurt, Senckenberg Biobank, ausgewählt.

### Fehlende Muskelinvasion in TCGA-Fällen

**Figure 2:**
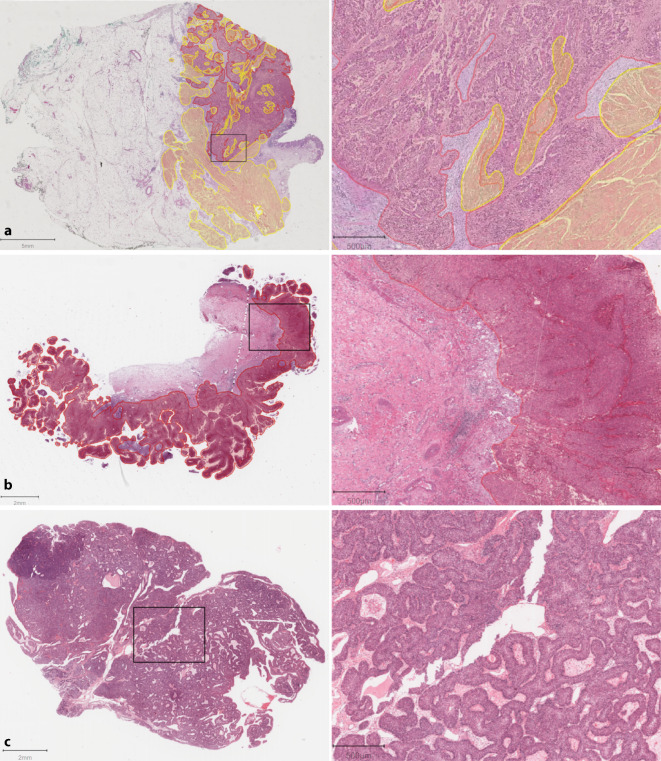

Uropathologen (Markus Eckstein, Peter J. Wild) begutachteten die HE-gefärbte Schnitte der frei zugänglichen Fälle (*N* = 386) der TCGA-BLCA-Kohorte im Hinblick auf Muskelinvasion und Bildqualität. Für das gesamte Manuskript ist „muskelinvasiv“ gleichzusetzen mit „detrusormuskelinvasiv“. Bei Uneinigkeiten hinsichtlich Muskelinvasion wurde ein Konsensusrating durch einen weiteren Pathologen (Henning Reis) gebildet. Für KI-Experimente inkludierten wir 292 Fälle, 94 Fälle wurden exkludiert, wovon mind. 69 Fälle keine histologische Muskelinvasion aufwiesen (siehe Tab. S1 und Abb. S1 im Onlinezusatzmaterial für exkludierte TCGA-Fälle). Gemäß der Originalpublikation wiesen 69 Fälle ohne histologisch verifizierbare Muskelinvasion ein klinisches Stadium T2–T4 auf [15]. Bereits Loeffler et al. bemerkten, dass die TCGA-BLCA-Kohorte teils histologische Schnitte von papillären Tumoren ohne muskelinvasive Anteile beinhaltet [12]. In Abb. 2 sind beispielhaft 3 Fälle – inklusive eines papillären Falls ohne Muskelinvasion (Abb. 2b) – der TCGA-BLCA-Kohorte abgebildet.

### Assoziation molekularer Subtypen und Überleben

Während Kamoun et al. eine signifikante Assoziation zwischen Konsensus-Subtypen und Gesamtüberleben beschrieben hatten, konnten wir für die inkludierten TCGA-Fälle (*N* = 292) keine signifikante Assoziation zwischen molekularen Konsensus-Subtypen und Gesamtüberleben erkennen (*p*-Wert multivariater Log-Rank-Test: 0,2) [8]. Kaplan-Meier-Plots für inkludierte Fälle und die gesamte TCGA-BLCA-Kohorte mit molekularen Subtypen sind in Abb. S2 im Onlinezusatzmaterial abgebildet.

### SIP-BLCA-Kohorte: Zuordnung RNA und Histologie

**Figure 3:**
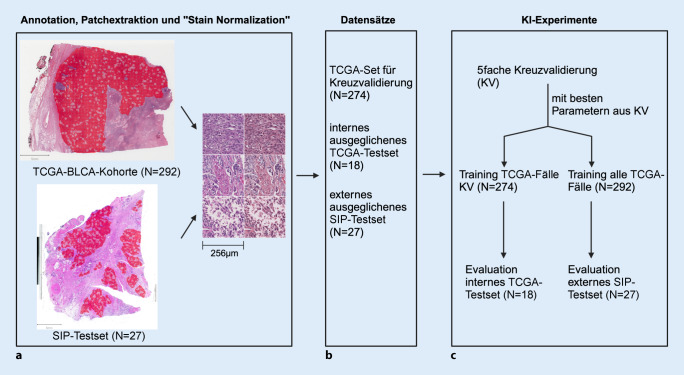

Für das ext. SIP-Testset wurden 103 von 181 MIUCs mit durchgeführter RNA-Extraktion, die von einer radikalen Zystektomie stammen, berücksichtigt [11]. Innerhalb der histologischen Schnitte wie auch innerhalb später erstellter Annotationen der SIP-Fälle (siehe Abb. 3a) konnte der Ort der RNA-Extraktion zugeordnet werden, während für die TCGA-BLCA-Kohorte eine RNA-Extraktion aus Frischgewebe und nicht aus den FFPE-Schnitten (FFPE, formalinfixiert und paraffineingebettet) für die histologischen Schnitte durchgeführt wurde [15]. Dies kann zu Probefehlern führen, da die annotierte Histomorphologie möglicherweise nicht mit dem bestimmten molekularen Subtyp identisch ist. Nach pathologischem Review auf Muskelinvasion im histologischen Schnitt durch einen Uropathologen (Henning Reis) wurden 27 Fälle für das nach Subtypen balancierte SIP-Testset pseudozufällig ausgewählt (Details und Einschlusskriterien SIP-Testset siehe Abb. S1 und „SIP-BLCA-Kohorte“ im Onlinezusatzmaterial). Mittels eines Tools von Kamoun et al. (siehe Onlinezusatzmaterial) ordneten wir für TCGA- und SIP-Fälle [10] molekulare Konsensus-Subtypen und Korrelationskoeffizienten den Subtypen zu [8].

### Annotationen TCGA-Fälle und ext. SIP-Testset

Tumorgewebe beider Kohorten wurde unter Supervision von mindestens einem Uropathologen in der Image-Viewer-Software „QuPath“ annotiert [2] und große Bereiche mit Nekrosen oder Artefakten wurden hierbei exkludiert. Pro Fall extrahierten wir bis zu 500 Patches aus den Annotationen mit der Größe 512 × 512 Pixel bei einer Auflösung von 0,5 µm pro Pixel, auf die eine „stain normalization“ [13] angewendet wurde (Abb. 3a). Für Testsetfälle (Abb. 3b) wurden zusätzlich alle Patches extrahiert.

## Künstliche Intelligenz in der Vorhersage molekularer Subtypen

### Experimente und Metriken

**Figure 4:**
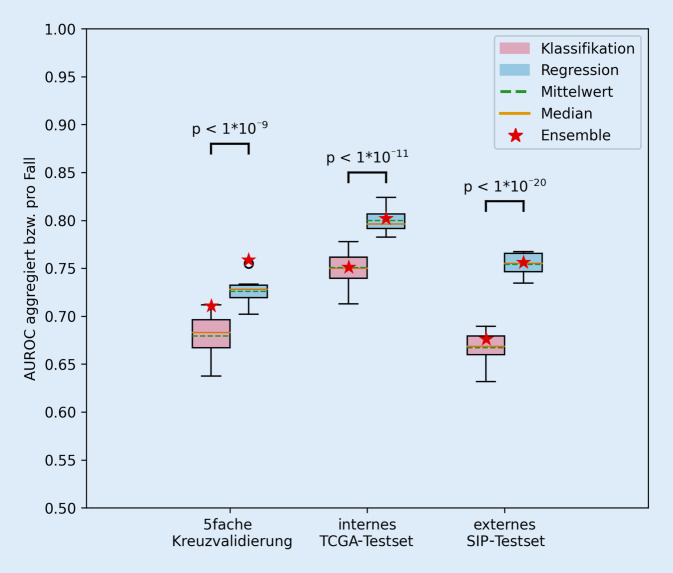

Auf „ImageNet“ vortrainierte ResNet18-Modelle wurden mit histologischen Schnitten der TCGA-BLCA-Kohorte trainiert, um die molekularen Subtypen „*luminal“*, „*Ba/Sq“* und „*stroma-rich“ *anhand der Histomorphologie vorherzusagen. Ein Ablauf der Experimente ist in Abb. 3c dargestellt. Inkludierte TCGA-Fälle wurden für eine Kreuzvalidierung (KV; *N* = 274) und ein nach Subtypen balanciertes internes (int.) Testset (*N* = 18) pseudozufällig aufgeteilt. Die besten Parameter aus einer 5fachen KV (z. B. Anzahl der Patches pro Fall, „random oversampling“ und Lernrate; siehe Abb. S3 und S4 im Onlinezusatzmaterial) wurden für ein erneutes KI-Training genutzt. Dazu wurde mit allen KV-Fällen trainiert und auf dem int. TCGA-Testset evaluiert bzw. mit allen inkludierten TCGA-Fällen (*N* = 292) trainiert und auf dem ext. SIP-Testset evaluiert (s. Abb. 3c). Experimente wurden jeweils 20-mal wiederholt und die KI-Modelle anhand durchschnittlicher Werte für „area under the receiver operating characteristic curve“ (AUROC) pro Fall (Berechnung mittels „macro-averaging“) und Genauigkeit pro Fall verglichen. Ebenso wurden die durch mehrere Modelle berechneten Metriken Ensemble-AUROC und Ensemblegenauigkeit berichtet. Signifikant bessere AUROC-Werte für KV und Testsets konnten dadurch erreicht werden, dass KI-Modelle mittels Regression auf den Pearson-Korrelationskoeffizienten pro Fall – z. B. „*luminal*“: 0,3, „*Ba/Sq*“: 0,5 und „*stroma-rich*“: 0,2 statt der Kategorie „*Ba/Sq*“ – trainiert wurden (Abb. 4). Pro Fall wurden Vorhersagen für zugehörige Patches aggregiert und durchschnittliche Vorhersagen berechnet. Die finale Subtypvorhersage pro Fall entsprach dem durchschnittlichen Korrelationskoeffizienten des Subtyps mit dem höchsten Wert. Als Luminal-Korrelationskoeffizient wurde pro Fall jeweils der Korrelationskoeffizient mit dem höchsten Wert unter den drei Luminal-Konsensus-Subtypen *(„luminal papillary“*, „*luminal non-specified“* und *„luminal unstable“*) ausgewählt. Neben ResNet18 experimentierten wir mit einer ResNet101-, ConvNeXt-Nano- und Eva-02-Small-Architektur (Vision Transformer), welche im externen SIP-Testset geringere Genauigkeiten pro Fall erreichten. Durch Vorhersage auf allen statt auf bis zu jeweils 500 Patches konnte die durchschnittliche Genauigkeit pro Fall mit einer ResNet18-Architektur für das ext. SIP-Testset von 0,41 auf 0,47 gesteigert werden. Im Onlinezusatzmaterial finden sich Details zu Metriken der Experimente (Tab. S2) sowie zu KI-Methoden, Software und Testsets. Beispiel-Quelltexte sind öffentlich abrufbar: https://github.com/alexandrastoll/MIBC_Molecular.

### Visualisierung intratumoraler Heterogenität

**Figure 5:**
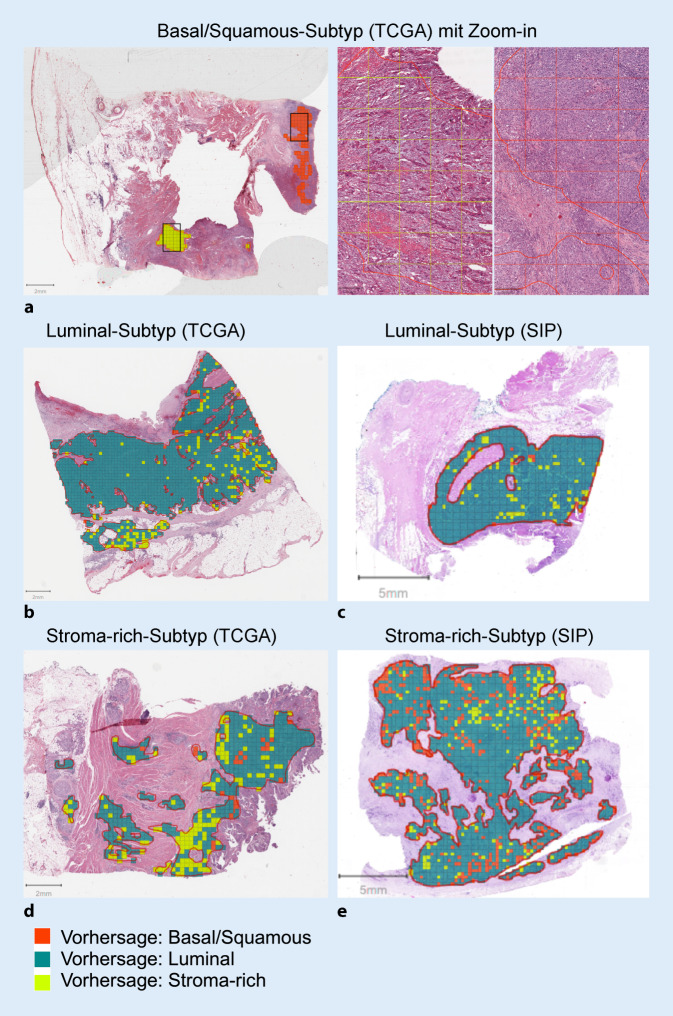

Zur Visualisierung einer intratumoralen molekularen Heterogenität wurden KI-Vorhersagen innerhalb annotierter Tumorareale des int. TCGA-Testsets und des ext. SIP-Testsets abgebildet (Abb. 5). In einem Fall mit molekularem Ba/Sq-Subtyp (Abb. 5a) wurde ein weniger dichtes Tumorareal mit umliegendem Stromagewebe (Zoom-in *links*) als ein Stroma-rich*-*Subtyp interpretiert. Histologisch konnte den beiden Arealen des Zoom-ins ein NOS-Typ des UCs zugeordnet werden. In Abb. S5 (Onlinezusatzmaterial) sind Beispiele aus der KV abgebildet mit jeweils niedriger und hoher vorhergesagter molekularer Heterogenität. Während unsere KI-Modelle in TCGA-Fällen insbesondere Ba/Sq- und Luminal-Subtypen relativ sicher vorhersagen konnten (s. Abb. S6 im Onlinezusatzmaterial), hatten die KI-Modelle Schwierigkeiten, Stroma-rich-Fälle zu identifizieren. Die korrekte Identifikation des molekularen Stroma-rich*-*Subtyps kann aber auch bei der Bulk-RNA-Sequenzierung mit Schwierigkeiten verbunden sein, da die Gensignaturen durch Stromazellen beeinflusst werden können [10].

### Relevante Merkmale für KI-Entscheidung

**Figure 6:**
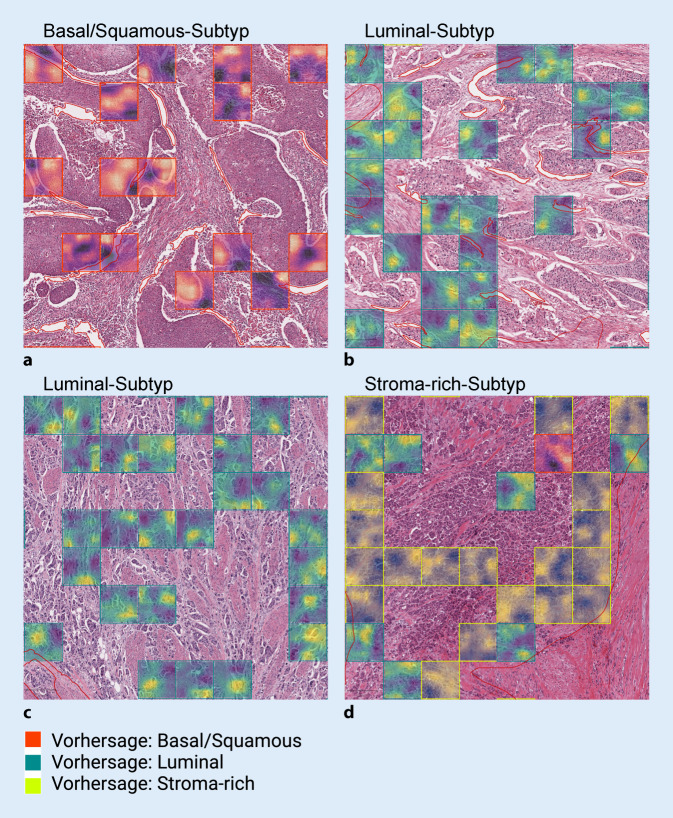

Um für die KI-Klassifikation relevante Areale zu visualisieren, setzten wir „Grad-CAM“ bei unserem KI-Modell ein (Abb. 6; [17]).

### Fälle ohne Muskelinvasion steigern Performance

Woerl et al. [20] und Angeloni et al. [1] zeigten, dass molekulare Subtypen in MIUCs bzw. in UCs des oberen Harntraktes mittels KI-Methoden anhand von HE-Schnitten vorhersagbar sind. Die Arbeit von Woerl et al. differenzierte 4 molekulare Subtypen und mehr Fälle (*N* = 363) der TCGA-BLCA-Kohorte wurden inkludiert, sodass unsere Ergebnisse nur bedingt vergleichbar sind. Woerl et al. erreichten durchschnittlich für alle Klassen AUROC-Werte von 0,89 und 0,87 („micro-“ und „macro-averaging“) für eine KV sowie AUROC-Werte von 0,85 (sowohl „micro-“ als auch „macro-averaging“) für ein ext. Testset (*N* = 16) [20]. Durch Zunahme von TCGA-Fällen ohne histologische Muskelinvasion, die auch von Woerl et al. [20] genutzt wurden (*N* = 65) und mehrheitlich einen luminal-papillären Subtyp aufwiesen (Tab. S1), steigerten wir durchschnittliche AUROC-Werte pro Fall (basierend auf „macro-averaging“) in der KV von 0,73 auf 0,79.

## Ausblick

Wir konnten zeigen, welche histomorphologischen Merkmale eine KI-Klassifikation von molekularen Subtypen mitbestimmen, und identifizierten mittels KI-Methoden eine teils intratumorale molekulare Heterogenität. Um verwendete KI-Methoden künftig zu validieren, sollten Tumorbereiche, die laut KI-Methoden verschiedene molekulare Subtypen aufweisen, zusätzlich basierend auf RNA-Extraktion subtypisiert werden. Eine Studienlimitation ist der begrenzte Augmentationsgebrauch: KI-Experimente mit weiteren Augmentationen (siehe Onlinezusatzmaterial) zeigten keine signifikante Verbesserung und blieben unberücksichtigt. Ebenso könnten neuere KI-Methoden, wie z. B. ein „self-supervised pretraining“, unsere Ergebnisse noch weiter verbessern [9].

## Fazit für die Praxis

- KI ist fähig, die intratumorale Heterogenität von molekularen Subtypen aufzuzeigen.
- Das maschinelle Lernen von Korrelationen von Subtypen (sog. Regression) erzielt eine höhere Genauigkeit als die alleinige Klassifikation molekularer Subtypen.
- Eine Überarbeitung der molekularen Konsensus-Subtypen für muskelinvasive Urothelkarzinome mit Fällen, die histologisch mindestens ein pT2-Stadium vorweisen, erscheint sinnvoll, sofern eine molekulare KI-Subtypisierung ihren Weg in die pathologische Routine finden soll.

### Supplementary Information





