# Supplementary material for: Histomolecular classification of urothelial carcinoma of the urinary bladder: From histological phenotype to genotype and back
Source: Pathologie (Heidelb). 2024 Jan 29;45(2):106–14. [Article in German] doi: 10.1007/s00292-024-01305-w (PMC10901926; doi:10.1007/s00292-024-01305-w)
Supplement: Supplementary file 2 [file 292_2024_1305_MOESM2_ESM.pdf]

Onlinezusatzmaterial mit Abb. S1-S6 und Tab. S2

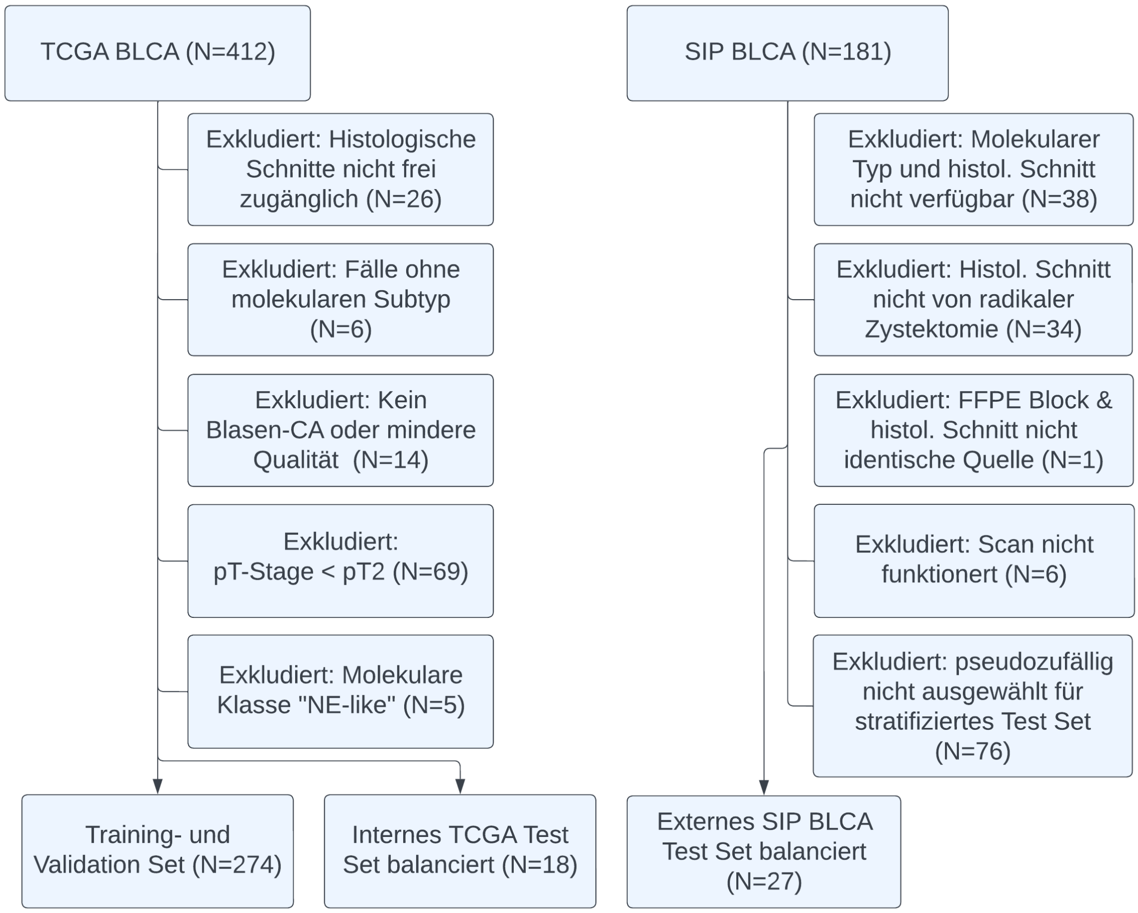

Abb. S1. Flow Chart für die Einschlusskriterien von inkludierten TCGA-Fällen und des SIP Test Sets. CA: Karzinom, „NE-like“: „neuroendocrine-like“, SIP: Dr. Senckenbergisches Institut für Pathologie.

TCGA BLCA Kohorte (The Cancer Genome Atlas Bladder Cancer Kohorte)

Von der gesamten TCGA BLCA Kohorte (N=412) luden wir 457 histologische Hämatoxylin-Eosin (HE) gefärbte Schnitte von 386 frei zugänglichen Fällen der TCGA BLCA Kohorte über das Genomic Data Commons (GDC) Portal herunter (https://portal.gdc.cancer.gov, Zugriff: 1.3.2022). Verfügbare klinisch-pathologische Informationen wurden von der supplementären Tabelle S1 von Robertson et al. [8] übernommen und auf weitere klinische Informationen zur TCGA BLCA Kohorte wurde über das cBio Cancer Genomics Portal zugegriffen

(https://www.cbioportal.org/study/summary?id=blca_tcga_pub_2017, Zugriff: 1.3.2022). Molekulare Konsensus-Subtypen wurden von der supplementären Tabelle S8 von der Publikation von Kamoun et al. übernommen, worin die TCGA BLCA Kohorte selbst zur Etablierung der Konsensus-Klassifikation genutzt wurde [4]. Wir verglichen die molekularen Konsensus-Klassenzuordnungen für die von Kamoun et al. analysierten 1750 muskelinvasiven UCs aus insgesamt 18 mRNA-Datensätzen mit sechs vorherigen molekularen Klassifikationen. Nach der Kombination der Fälle mit zugeordneten *„luminal“* Konsensus-Subtypen zu einer gemeinsamen Gruppe wurde festgestellt, dass es Überlappungen zwischen dieser Gruppe und Subtypen früherer Klassifikationen gab. Aufgrund dieser Überlappungen und weil es in der verwendeten TCGA BLCA Kohorte nur wenige Fälle des „*luminal non-specified*“- und des *„neuroendocrine-like“*-Subtyps gab, wurden entsprechende „*luminal*“-Konsensus-Fälle für nachfolgende Experimente als ein gemeinsamer *„luminal“*-Subtyp behandelt – was eine Vereinfachung darstellt – und „*neuroendocrine-like“*-Fälle wurden von der weiteren Analyse ausgeschlossen. Ausgehend von den in Kamoun et al. [4] berücksichtigten 406 Fällen der TCGA BLCA Kohorte wurden von uns 114 TCGA-Fälle exkludiert, von denen 70 Fälle laut Robertson et al. eine papilläre Morphologie aufwiesen [8] und von denen 52 Fällen wiederum – nach Kamoun et al. – ein *„luminal papillary“* Subtyp zugeordnet werden konnte [4].

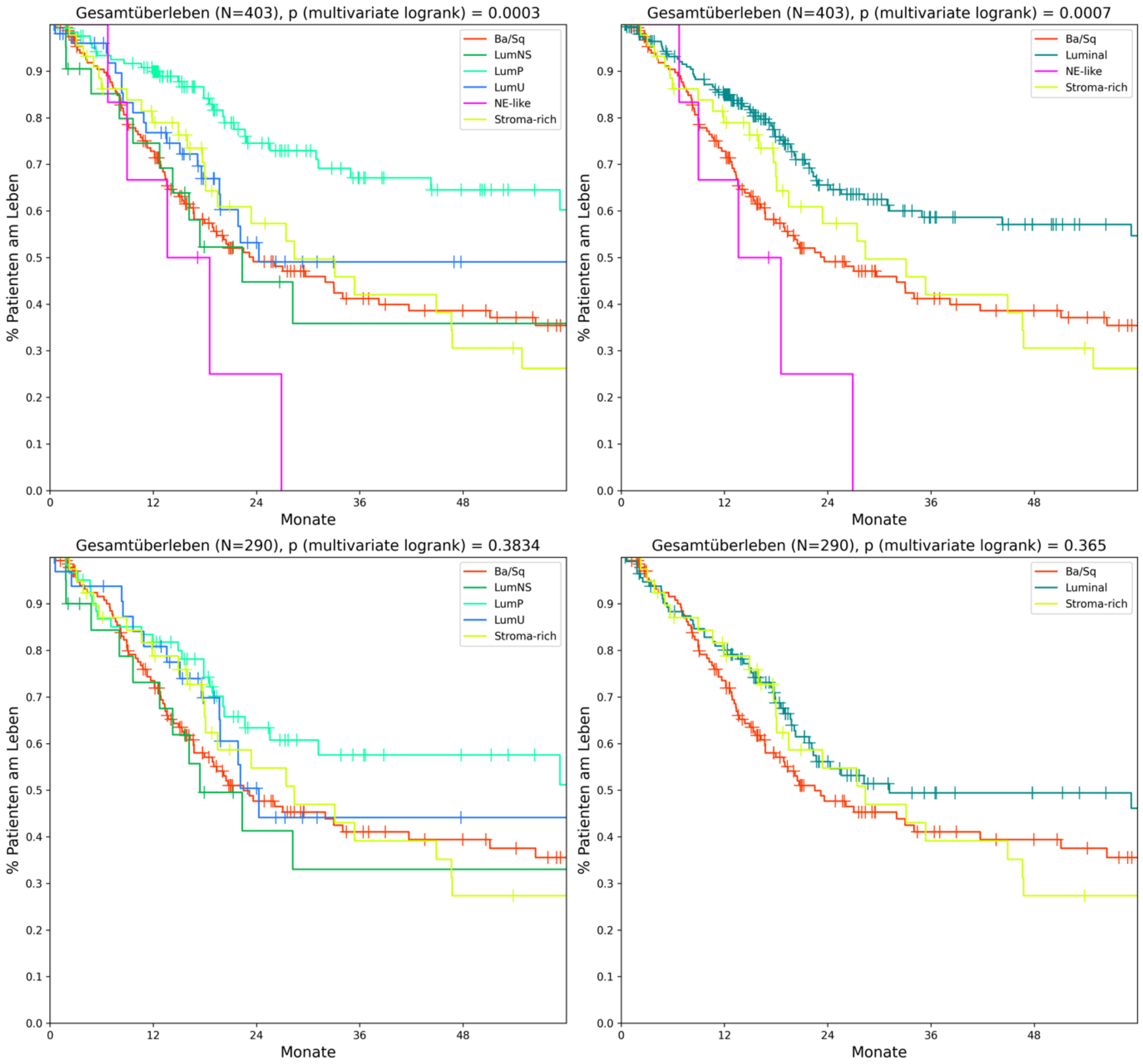

Abb. S2. Kaplan-Meier Plots für alle TCGA BLCA Fälle mit vorhandenen molekularen Konsensus-Subtypen und verfügbaren Überlebensdaten (oben) und Kaplan-Meier Plots für nur nach histologischem Review inkludierte TCGA BLCA Fälle mit verfügbaren Überlebensdaten (unten). Links sind jeweils alle in vorhandenen Datensätzen inkludierte Konsensus-Subtypen zugeordnet, während rechts von uns verwendete molekulare Subtypen abgebildet sind. Für inkludierte Fälle kann keine signifikante Assoziation zwischen Gesamtüberleben und molekularen Konsensus-Subtypen festgestellt werden.

SIP BLCA Kohorte (Dr. Senckenbergisches Institut für Pathologie Bladder Cancer Kohorte)

Entsprechende Kohorte wurde bereits von Koll et al. publiziert [6]. Von 181 muskelinvasiven Urothelkarzinom-Fällen wurden 143 Fälle berücksichtigt, für welche sowohl HE-Schnitte als auch RNA-Sequenzierungsdaten vorhanden waren. Für die RNA-Extraktion der hier verwendeten Fälle (durch Florestan J. Koll) waren das MACE

Gene Panel von GenXPro und das HTG Transcriptome Panel von HTG Molecular Diagnostics verwendet worden. Die schriftliche Einwilligung aller Patienten wurde eingeholt, und die Studie wurde von den institutionellen Prüfgremien des UCT Frankfurt-Marburg und der Ethikkommission des Universitätsklinikums Frankfurt genehmigt (Projektnummer: SUG-6-2018 und UCT-53-2021). Die Studie wurde gemäß den lokalen und nationalen Vorschriften und der Deklaration von Helsinki durchgeführt. Es wurden 39 Fälle – wovon ein Teil als Reserve diente – stratifiziert nach molekularen Konsensus-Subtypen pseudozufällig ausgewählt (siehe Details verwendete Software unten). Nach pathologischem Review wurden von den anfangs 27 ausgewählten Fällen zwei Fälle ohne sichtbare Muskelinvasion im histologischen Schnitt durch zwei Fälle aus der Reserve ersetzt.

Software

Mittels eines Tools von Kamoun et al. (Link: https://github.com/cit-bioinfo/consensusMIBC, Zugriff: 9.8.2023) wurden für die TCGA-Fälle und die SIP-Fälle [5] molekulare Konsensus-Subtypen und Korrelationskoeffizienten zu den Subtypen zugeordnet [4].

Tumorgewebe wurde mit der Image Viewer Software „QuPath“ (Version 0.3.2) annotiert und Patches wurden mittels eines Groovy-Skriptes extrahiert [1].

KI-Experimente wurden unter Python (Version 3.1.12) und folgenden Python-Packages durchgeführt:

„PyTorch“ (Version 2.0), „Fast.ai“ [3] (Version 2.7.12), „Albumentations“ [2] (Version 1.3), „imblearn“ (Version 0.9.1) und „Weights & Biases“ (Version 0.13.5). Vortrainierte ResNet18“ Architekturen wurden von dem Python-Package „Pytorch image models“ (timm) (Version: 0.8.9) genutzt.

Folgende statistische Tests mit entsprechenden Python-Packages wurden durchgeführt: T-Tests auf Unabhängigkeit wurden mit dem „Scipy.stats“-Software Package (Version 1.9.2) und Logrank-Tests wurden mit dem „Lifeline.statistics“-Package durchgeführt (Version: 0.27.4). In KI-Experimenten wurden weitestgehend keine Random-Seeds genutzt, um Experimente möglichst zufällig ablaufen zu lassen. Test Sets wurden pseudozufällig mittels „Sample“-Methode des „Pandas"-Packages (Version 1.5.2) jeweils pro Subtyp ausgewählt. *„Luminal“*-Fälle für die Test Sets wurden zu gleichen Anteilen pseudozufällig aus *„luminal papillary“, „luminal non-*

specified" und *„luminal unstable"* zusammengesetzt. Für das Training auf Korrelationen im Rahmen einer Regression, wurde Code von dem folgenden Tutorial übernommen: https://walkwithfastai.com/Multi_Point_Regression, Zugriff: 26.3.2023). Für das „Albumentations"-Package wurde Code des folgenden Tutorials modifiziert übernommen: https://docs.fast.ai/tutorial.albumentations.html, Zugriff: 5.9.23).

KI-Algorithmen

Transfer-Learning: Für eine Epoche wurde nur die neue, zufällig initialisierte letzte „Layer" des vortrainierten Netzwerks trainiert, anschließend wurde das gesamte Netzwerk zusätzlich für eine Epoche trainiert. Eine „Batchsize" von 60 wurde verwendet.

Optimizer: Als Optimizer wurde „AdamW" (Adam with decoupled weight decay) [7] genutzt.

Lernraten-Policy: Es wurde eine sogenannte „1cycle"-Policy [9] mit einer Basis-Lernrate von 0.002 verwendet. Es wurde zusätzlich mit einer Basis-Lernrate von 0.001 und 0.004 experimentiert (**Abb.S4**).

Augmentationen: Es wurden eine zufällige Rotation um 90° sowie vertikales und horizontales Spiegeln im Training angewandt. Es wurde ebenso mit Augmentationen wie "Stain Augmentations" (im „HED-Space" und „HSV-Space") [10], „MixUp", „GridDistortion" und „ElasticTransformation" experimentiert, ohne eindeutige Verbesserungen zu erzielen. Entsprechende Experimente wurden daher nicht aufgenommen.

Hardware

KI-Berechnungen wurden auf einer „RTX3090"-Grafikkarte mit 24 GB VRam durchgeführt.

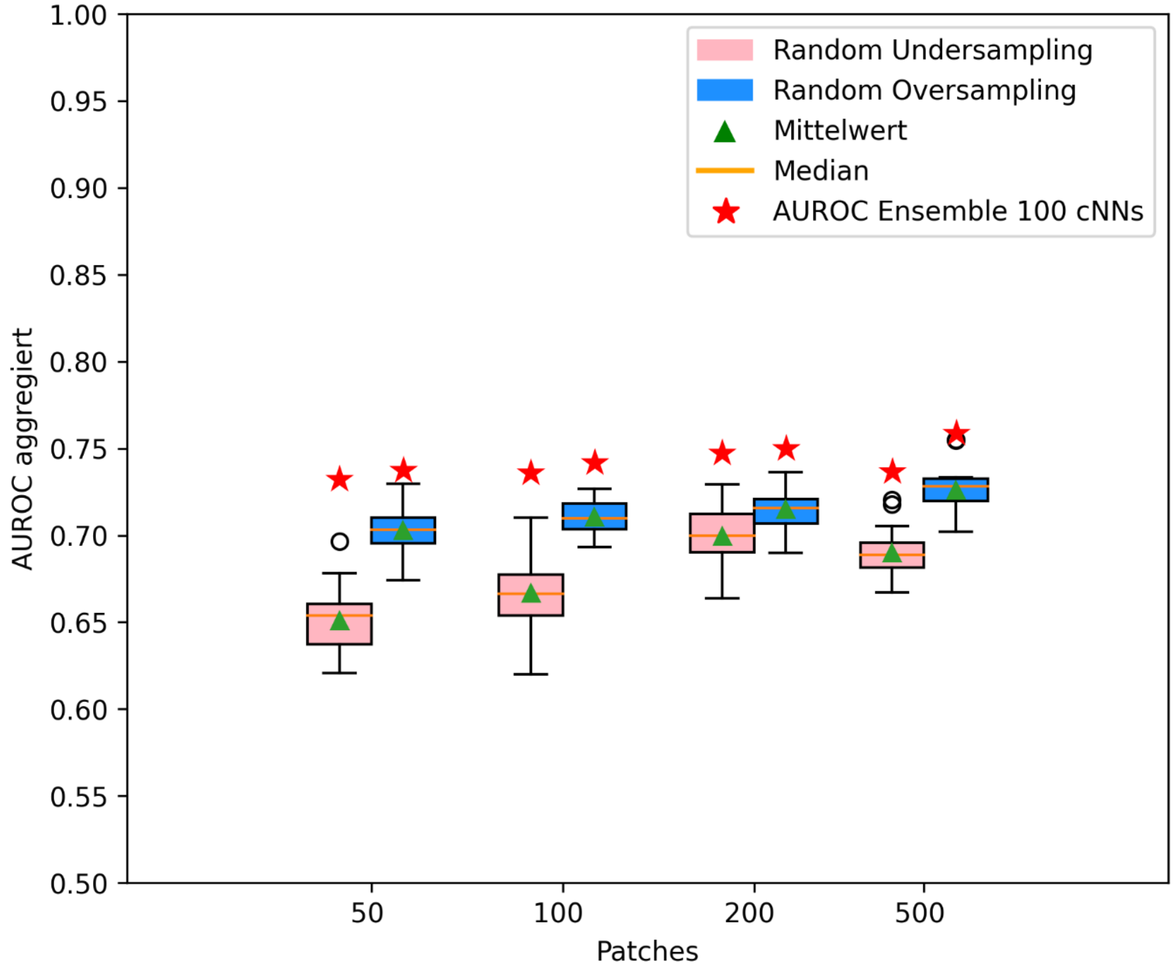

Abb. S3. Für 50, 100, 200 und 500 verwendete Patches pro Fall wurde jeweils ein „Random Oversampling” und ein „Random Undersampling” durchgeführt. Ein „Random Oversampling“ mit 500 Patches pro Fall erreichte die höchsten AUROC-Werte aggregiert bzw. pro Fall, sodass diese beiden Einstellungen für ein späteres, erneutes Training auf mehr Fällen und zur Vorhersage auf den Test Sets verwendet wurde.

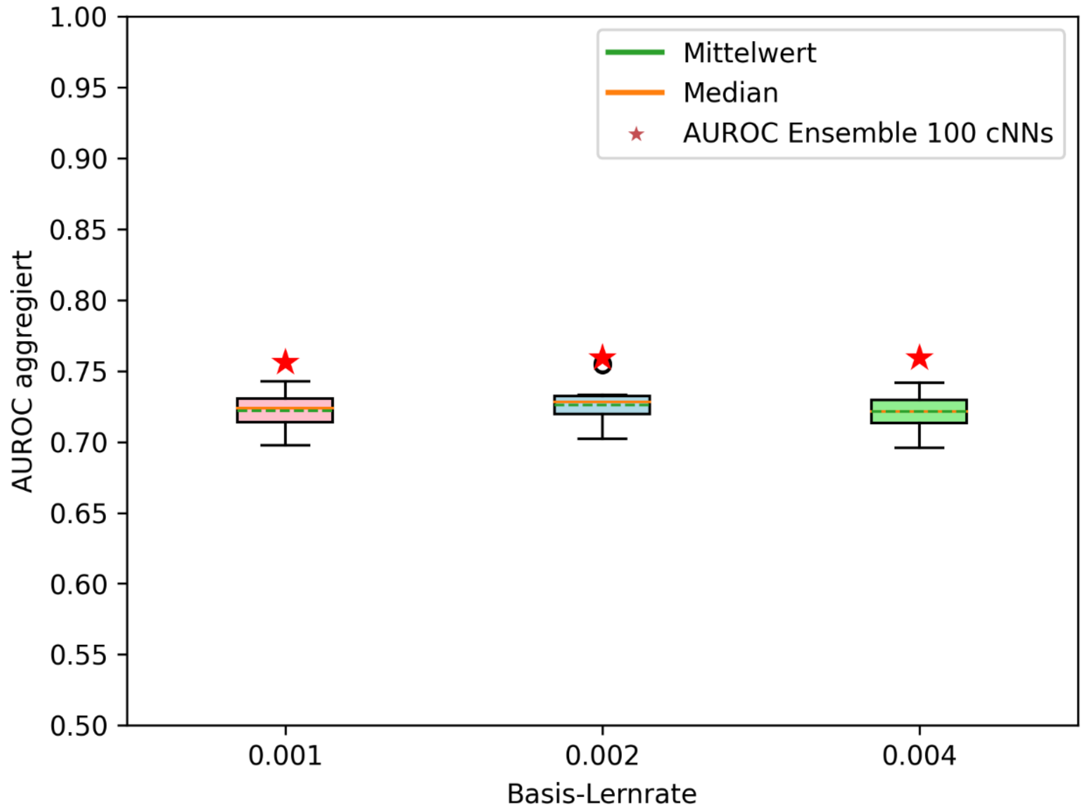

Abb. S4. Für 500 Patches in Kombination mit „Random Oversampling“ wurde mit verschiedenen Basis-Lernraten experimentiert. Für eine Basis-Lernrate von 0.002 wurde ein höchster durchschnittlicher AUROC-Wert aggregiert bzw. pro Fall von 0.7261 (basierend auf „Macro-Averaging“) erreicht, während für eine Basis-Lernrate von 0.004 ein leicht höherer „Ensemble-AUROC“-Wert erreicht wurde (0.7581 vs. 0.7578 bei einer Basis-Lernrate von 0.002). So wurde eine Basis-Lernrate von 0.002 für ein späteres erneutes Training auf mehr Fällen und zur Vorhersage auf den Test Sets verwendet. Eine Basis-Lernrate wird innerhalb der „1cycle"-Policy verwendet.

Tab. S2. Ergebnisse KI-Experimente für Regression und Klassifikation für Kreuzvalidierung und Test Sets. Sofern nicht anders spezifiziert, wurden jeweils bis zu 500 Patches pro Fall in Training und Validierung genutzt. Eine „ResNet18“-Architektur mit Regression war komplizierteren Architekturen in Bezug auf Genauigkeit pro Fall für das ext. SIP Test Set überlegen. Durch Validation auf alle Patches pro Fall bei gleichzeitigem Training auf bis zu jeweils 500 Patches, konnte die durchschnittliche Genauigkeit pro Fall von ca. 0.41 auf 0.47 für das ext. SIP Test Set gesteigert werden. Metriken wurden bis auf „Ensemble“-Metriken als durchschnittliche Metriken nach 20 Wiederholungen mit Standardabweichung angegeben. *Skalierung der Patches zu 256x256 Pixeln statt 224x224 Pixeln mit der „Resize“-Methode von „fast.ai“.

Datensatz/Einstellungen	AUC Patch	AUC Fall	Genauigkeit Patch	Genauigkeit Fall	Ensemble AUC Fall	Ensemble Genauigkeit Fall
5-fache KV, Klassif., ResNet18	0.61 ± 0.01	0.68 ± 0.01	0.43 ± 0.01	0.48 ± 0.02	0,56	0,41
5-fache KV, Regr., ResNet18	0.66 ± 0.01	0.73 ± 0.01	0.47 ± 0.01	0.53 ± 0.02	0,76	0,65
5-fache KV, Regr., ResNet18*	0.66 ± 0.01	0.72 ± 0.01	0.47 ± 0.01	0.52 ± 0.02	0,76	0,64
5-fache KV, Regr., ResNet101	0.63 ± 0.01	0.7 ± 0.01	0.44 ± 0.01	0.52 ± 0.02	0,73	0,61
5-fache KV, Regr., ConvNeXt-Nano	0.68 ± 0.01	0.74 ± 0.01	0.5 ± 0.01	0.57 ± 0.02	0,77	0,66
5-fache KV, Regr., Eva-02-Small	0.68 ± 0.01	0.73 ± 0.01	0.49 ± 0.01	0.55 ± 0.02	0,77	0,66
int. TCGA Test Set, Klassif., ResNet18	0.68 ± 0.01	0.76 ± 0.01	0.51 ± 0.01	0.66 ± 0.02	0,75	0,67
int. TCGA Test Set, Regr., ResNet18	0.74 ± 0.01	0.8 ± 0.01	0.53 ± 0.01	0.54 ± 0.02	0,8	0,56
int. TCGA Test Set, Regr., ResNet18*	0.74 ± 0.01	0.81 ± 0.01	0.54 ± 0.01	0.54 ± 0.02	0,8	0,56
int. TCGA Test Set, Regr., ConvNetX Nano	0.76 ± 0.01	0.81 ± 0.01	0.54 ± 0.01	0.56 ± 0.02	0,8	0,5
int. TCGA Test Set, Regr., ResNet101	0.71 ± 0.01	0.79 ± 0.01	0.51 ± 0.01	0.49 ± 0.02	0,79	0,44
int. TCGA Test Set, Eva-02-Small	0.76 ± 0.01	0.81 ± 0.01	0.56 ± 0.01	0.57 ± 0.02	0,8	0,56
int. TCGA Test Set, Regr., ResNet18, alle Patches	0.74 ± 0.01	0.79 ± 0.01	0.54 ± 0.01	0.5 ± 0.02	0,79	0,44
ext. SIP Test Set, Klassif., ResNet18	0.59 ± 0.01	0.67 ± 0.01	0.39 ± 0.01	0.38 ± 0.02	0,67	0,37
ext. SIP Test Set, Regr., ResNet18	0.67 ± 0.01	0.75 ± 0.01	0.41 ± 0.01	0.41 ± 0.02	0,76	0,41
ext. SIP Test Set, Regr., ResNet18*	0.67 ± 0.01	0.77 ± 0.01	0.42 ± 0.01	0.42 ± 0.02	0,77	0,44
ext. SIP Test Set, Regr., ResNet101	0.63 ± 0.01	0.74 ± 0.01	0.39 ± 0.01	0.36 ± 0.02	0,76	0,37
ext. SIP Test Set, Regr., ConvNeXt Nano	0.63 ± 0.01	0.7 ± 0.01	0.36 ± 0.01	0.33 ± 0.02	0,71	0,33
ext. SIP Test Set, Regr., Eva-02-Small	0.68 ± 0.01	0.79 ± 0.01	0.42 ± 0.01	0.37 ± 0.02	0,8	0,37
ext. SIP Test Set, Regr., ResNet18, alle Patches	0.65 ± 0.01	0.74 ± 0.01	0.42 ± 0.01	0.47 ± 0.02	0,74	0,48
5-fache KV, Regr., ResNet18, pT1+, N=357	0.71 ± 0.01	0.79 ± 0.01	0.51 ± 0.01	0.58 ± 0.02	0,81	0,69

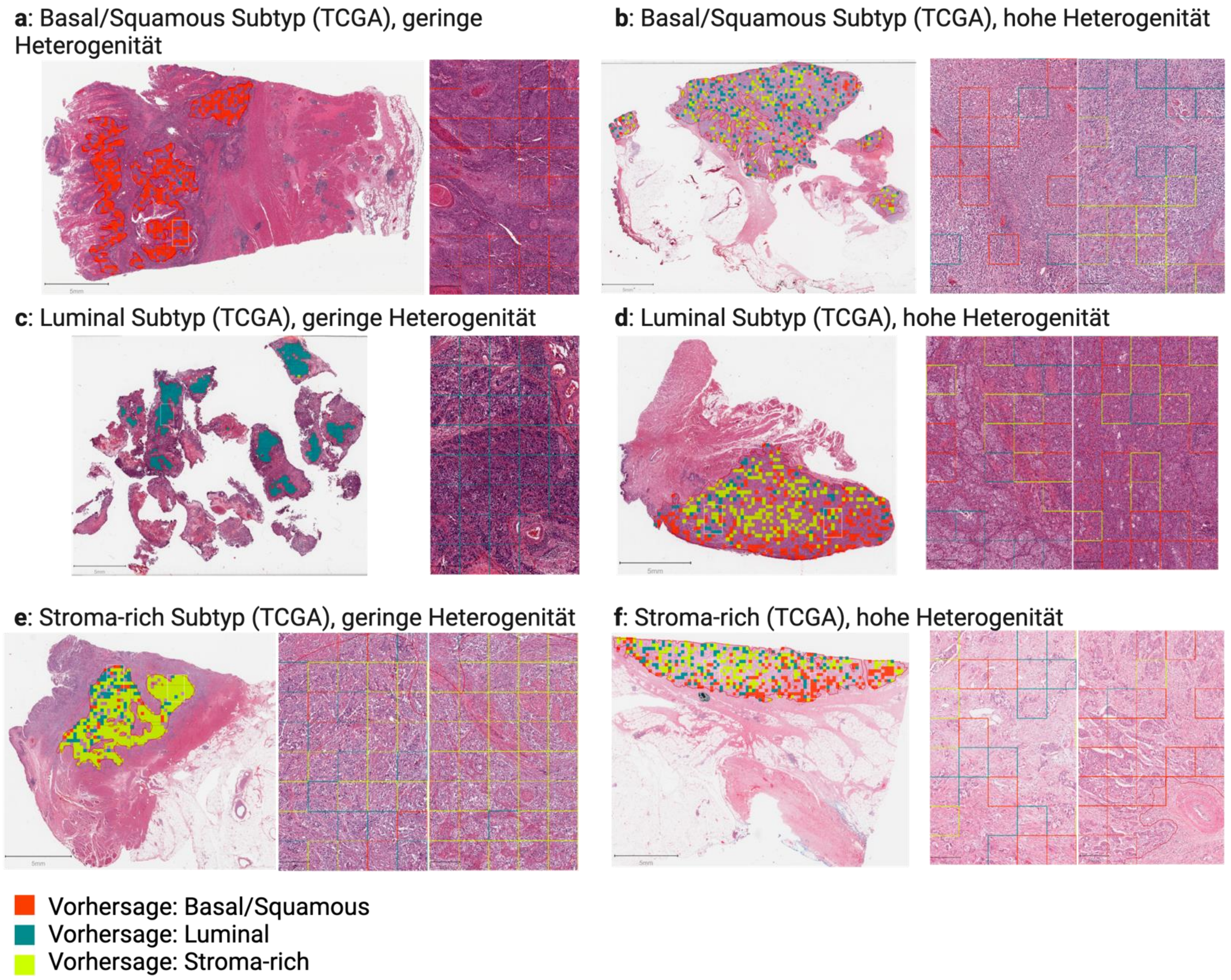

Abb. S5. Ensemble-Vorhersagen für sechs TCGA BLCA Fälle aus der Kreuzvalidierung mit jeweils Zoom-In rechts (Maße vergrößerter Areale je 1280x2048µm). Erstellt mit BioRender.com

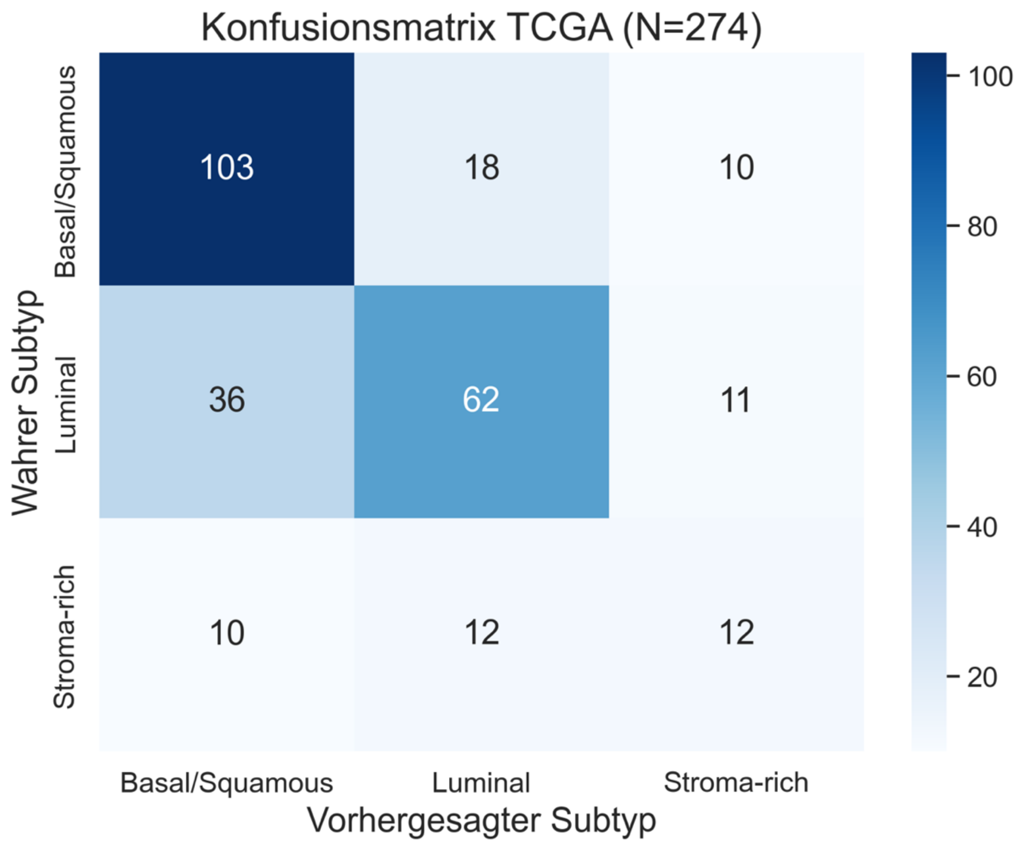

Abb. S6. Konfusionsmatrix zeigt vorhergesagte und eigentliche molekulare Subtypen für die 274 Fälle, die Teil der Kreuzvalidierungs-Experimente waren. Vorhersagen stammen von einem “ResNet18”-Ensemble, das aus 100 einzelnen Modellen von 20 Wiederholungen einer 5-fachen Kreuzvalidierung berechnet wurde. Während Fälle mit *„basal/squamous-“* und *„luminal“*-Subtyp sehr gut und mit moderater Genauigkeit vorhergesagt werden können, hatte unser trainiertes KI-Modell Schwierigkeiten, einen *„stroma-rich“*-Subtyp vorherzusagen.

Literatur:

1. Bankhead P, Loughrey MB, Fernández JA et al. (2017) QuPath: Open source software for digital pathology image analysis. Scientific reports 7:1-7
2. Buslaev A, Iglovikov VI, Khvedchenya E et al. (2020) Albumentations: Fast and Flexible Image Augmentations. Information 11:125
3. Howard J, Gugger S (2020) Fastai: A Layered API for Deep Learning. Information 11
4. Kamoun A, De Reynies A, Allory Y et al. (2020) A Consensus Molecular Classification of Muscle-invasive Bladder Cancer. Eur Urol 77:420-433
5. Koll FJ, Doring C, Olah C et al. (2023) Optimizing identification of consensus molecular subtypes in muscle-invasive bladder cancer: a comparison of two sequencing methods and gene sets using FFPE specimens. BMC Cancer 23:504
6. Koll FJ, Schwarz A, Kollermann J et al. (2022) CK5/6 and GATA3 Defined Phenotypes of Muscle-Invasive Bladder Cancer: Impact in Adjuvant Chemotherapy and Molecular Subtyping of Negative Cases. Front Med (Lausanne) 9:875142
7. Loshchilov I, Hutter F (2017) Decoupled weight decay regularization. arXiv preprint arXiv:1711.05101
8. Robertson AG, Kim J, Al-Ahmadie H et al. (2017) Comprehensive Molecular Characterization of Muscle-Invasive Bladder Cancer. Cell 171:540-556 e525
9. Smith LN, Topin N (2017) Super-convergence: Very fast training of neural networks using large learning rates. In, p 1-18
10. Tellez D, Litjens G, Bandi P et al. (2019) Quantifying the effects of data augmentation and stain color normalization in convolutional neural networks for computational pathology. Med Image Anal 58:101544
